# Spontaneous Closure of a Foveal Neurosensory Dehiscence Beneath an Intact Internal Limiting Membrane Following Spontaneous Premacular Haemorrhage: A Case Report

**DOI:** 10.1155/crop/4227953

**Published:** 2026-11-14

**Authors:** Anthony Daher, Nour Jabbour, Yehya Tlaiss, Bassam Barakat

**Affiliations:** ^1^ Department of Ophthalmology University of Balamand Beirut Lebanon balamand.edu.lb; ^2^ Department of Ophthalmology Mount Lebanon Hospital Beirut Lebanon

**Keywords:** foveal dehiscence, internal limiting membrane, lamellar macular hole, optical coherence tomography, premacular haemorrhage, spontaneous closure, subinternal limiting membrane haemorrhage, Valsalva retinopathy

## Abstract

**Background:**

Premacular haemorrhage, whether it follows a Valsalva manoeuvre or arises without one, usually lies beneath the internal limiting membrane (ILM). Foveal dehiscence is a rare sequela. The only reported case of a defect arising spontaneously beneath a preserved ILM enlarged and never closed; defects meeting criteria for a true full‐thickness macular hole have either been unmasked by intervention or have needed vitrectomy.

**Case Presentation:**

A 51‐year‐old hypertensive man presented with a sudden central scotoma of the left eye and vision of counting fingers. No precipitating Valsalva manoeuvre could be elicited. A dense premacular haemorrhage overlay the fovea; OCT showed a hyperreflective dome with dense posterior shadowing (central subfield thickness 571 *μ*m; fellow eye 223 *μ*m). Fluorescein angiography showed blocking by the haemorrhage, with no macroaneurysm, occlusion or neovascular lesion in the visible retina, though a macroaneurysm beneath the blood could not be excluded angiographically. He was observed. At 8 weeks, with the blood cleared, acuity was 20/60 with eccentric fixation and OCT disclosed a foveal defect running from the inner retina to the retinal pigment epithelium with the external limiting membrane and ellipsoid zone interrupted, but the ILM intact and reapposed. The lesion therefore did not meet International Vitreomacular Traction Study Group criteria for a full‐thickness macular hole. At 22 weeks, it had closed spontaneously, with a restored foveal contour and a vertical hyperreflective column consistent with glial repair. Best‐corrected acuity was 20/20.

**Conclusions:**

A foveal neurosensory dehiscence beneath a preserved ILM can close on its own after a premacular haemorrhage, with full visual recovery and no laser, surgery or drug treatment. In the only comparable published case, the ILM stayed detached, thickened and tractional and the defect enlarged; in ours, it was reapposed and the defect closed. What happens to these defects may therefore depend on whether traction from the distended ILM is relieved or maintained a distinction that may identify eyes worth observing rather than operating.

## 1. Background

Premacular haemorrhage classically follows a Valsalva manoeuvre, in which a sudden rise in intrathoracic pressure against a closed glottis ruptures superficial perifoveal capillaries [1]; it may also arise with no identifiable precipitant. Whether the blood lies beneath the ILM, the posterior hyaloid, or both can be settled on OCT only when the two membranes are separately resolved [2, 3], though a sub‐ILM location is now regarded as the more common [2, 3].

The natural history is usually benign and observation is the default [4]. Nd:YAG membranotomy and vitrectomy are reserved for dense, central or persistent haemorrhages, because prolonged contact between blood and retina risks photoreceptor toxicity, epiretinal membrane and foveal dehiscence [4, 5].

Foveal dehiscence after such a haemorrhage is rare, and the reported cases differ in both anatomy and outcome. Ulbig et al. found a macular hole after Nd:YAG drainage of subhyaloid blood [6]; Kim et al. described one that appeared after vitrectomy and needed a second operation [7]; Suren et al. reported a full‐thickness hole a week after membranotomy that later closed on its own [8]. Most recently, a full‐thickness hole arose spontaneously as a haemorrhage cleared, failed to close over 12 weeks, and was repaired by vitrectomy with ILM peeling and gas [9].

One report is anatomically different from all of these and is our direct comparator. Xie et al. followed a 34‐year‐old man who bled after push‐ups and was observed. As the blood resorbed, OCT showed the neurosensory retina discontinuous at the fovea with the ILM itself intact, what they called an outer retinal lamellar macular hole. The ILM stayed detached and thickened and pulled on the fovea. The defect measured 117 *μ*m at 3 months, 296 *μ*m at 9, and 288 *μ*m at 17. It never closed [10].

We describe the same anatomy, a foveal neurosensory dehiscence beneath a preserved ILM, appearing as a premacular haemorrhage cleared but with the opposite result: spontaneous closure by glial bridging, vision from counting fingers to 20/20, and no treatment of any kind. We are not aware of a previous report of this. We think the difference comes down to what the ILM did afterwards.

## 2. Case Presentation

A 51‐year‐old man presented in January 2026 with a sudden, painless central scotoma of the left eye of approximately 3 hours′ duration. He had treated systemic arterial hypertension. No precipitating event could be identified: on direct and repeated questioning the patient recalled no lifting, coughing, vomiting, straining or other Valsalva‐type manoeuvre preceding the onset of symptoms. There was no history of ocular trauma, intraocular surgery, high myopia or anticoagulant use, and no personal or family history of bleeding diathesis.

Uncorrected visual acuity was 20/25 in the right eye and counting fingers in the left.

Pupils were reactive, no RAPD, IOP normal in both eyes; anterior segment and lens are also normal.

Dilated fundoscopy of the left eye showed a dense, well‐circumscribed premacular haemorrhage overlying the fovea with an associated intraretinal component (Figure 1A). The right fundus was normal, with no hypertensive retinopathy and no macroaneurysm (Figure 1E).

**Figure 1:**
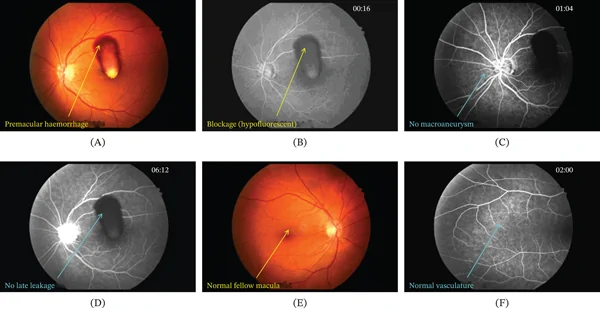
Fluorescein angiography (Kowa VX‐20), January 26, 2026. (A) Colour photograph of the left eye: a dense, keel‐shaped premacular haemorrhage overlies the fovea. (B–D) Left eye at 00:16, 01:04 and 06:12: the haemorrhage stays densely hypofluorescent throughout, its outline reproducing the lesion seen clinically—blocking rather than a filling defect—with subtle hyperfluorescence at its margins. In the retina not obscured by blood, there is no aneurysmal dilatation, capillary nonperfusion, venous staining or late leakage; because the fovea itself is blocked, a macroaneurysm lying beneath the blood cannot be excluded from these images alone. (E,F) Right eye: normal fundus and symmetrical filling. Juxtapapillary and optic nerve head staining were present in both eyes. Arrows mark the structures described; blue arrows in (C), (D) and (F) indicate the arcade arterioles and macula, in visible retina, where no abnormality was seen.

Review at one and 3 weeks showed no change: visual acuity remained counting fingers and the haemorrhage was unchanged in extent, with no dependent layering.

OCT (Optovue iVue, 6 × 6 mm Retina Map) on January 14, 2026 showed a large, dome‐shaped, homogeneously hyperreflective lesion centred on the fovea, elevating the inner retinal surface and shadowing the outer retina and retinal pigment epithelium. Central subfield thickness was 571 *μ*m (superior 606, inferior 333, nasal 403, temporal 436 *μ*m) against 223 *μ*m in the fellow eye. The foveal architecture was not assessable (Figure 2A).

**Figure 2:**
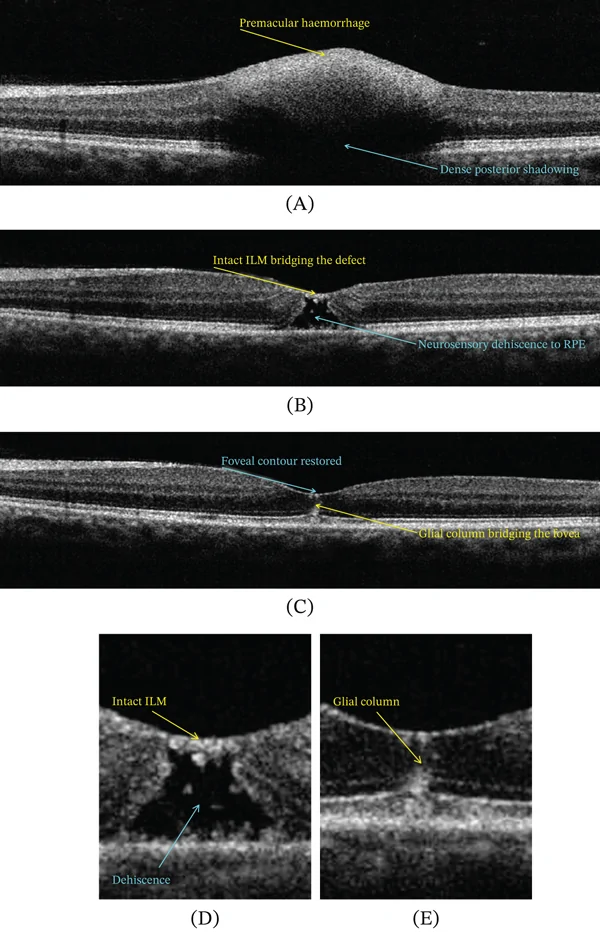
Serial spectral‐domain OCT of the left macula (Optovue iVue, 6 × 6 mm retina map; horizontal B‐scans through the fovea). (A) January 14, 2026: a hyperreflective dome occupies the fovea and shadows the outer retina and retinal pigment epithelium; the foveal architecture is not assessable. Central subfield thickness (CST) 571 *μ*m. (B) March 2, 2026: with the blood resorbed, a hyporeflective cavity occupies the fovea and extends to the retinal pigment epithelium, interrupting the outer nuclear layer, external limiting membrane and ellipsoid zone —but a thin continuous hyperreflective band, the preserved ILM, runs uninterrupted across its inner aspect. CST 272 *μ*m. (C) June 8, 2026: the cavity has resolved, the foveal contour is restored (CST 257 *μ*m), and a vertical hyperreflective column spans the retina at the site of the former defect, consistent with glial repair. (D, E) Magnified views of (B) and (C). Yellow arrows mark the preserved ILM (B,D) and the glial column (C,E); blue arrows mark the posterior shadowing (A), the dehiscence (B) and the restored contour (C).

Fluorescein angiography (Kowa VX‐20) on 26 January 2026 showed hypofluorescence corresponding to blocking by the haemorrhage, with subtle foveal and perifoveal hyperfluorescence and juxtapapillary and optic nerve head staining in both eyes. In the retina not obscured by blood, there was no aneurysmal filling, no arteriolar abnormality, no capillary nonperfusion or venous staining, and no late leakage (Figure 1). Because the haemorrhage blocked the fovea itself, a macroaneurysm lying directly beneath it could not be excluded on angiography alone. Systemic workup was performed and was unremarkable: CBC, platelets, coagulation profile, Hba1C and blood pressure measurement were within normal.

## 3. Emergence of the Foveal Defect

By March 2, 2026, approximately 7 weeks after onset, the patient reported improvement. Uncorrected visual acuity in the left eye was 20/60, achieved with eccentric fixation. The premacular haemorrhage had cleared almost completely.

OCT now disclosed a foveal defect, with central subfield thickness fallen to 272 *μ*m. A hyporeflective cavity occupied the fovea, running from the inner retina to the retinal pigment epithelium, with the outer nuclear layer, external limiting membrane and ellipsoid zone interrupted across the foveal centre. The internal limiting membrane, however, remained intact, bridging the inner aspect of the cavity as a continuous thin hyperreflective band between the two edges of the defect (Figure 2B,D). The lesion therefore spared the ILM and did not satisfy International Vitreomacular Traction Study Group criteria for a full‐thickness macular hole, which require a defect from the ILM to the retinal pigment epithelium [11]. International Vitreomacular Traction Study Group criteria for a full‐thickness macular hole, which require a defect extending from the ILM to the retinal pigment epithelium [11].

Calliper measurement was not available on the platform, so dimensions are described rather than measured: the defect was confined to the central fovea. The edges showed no cystoid degeneration, and there was no epiretinal membrane, subretinal fluid or vitreomacular traction. Critically, the overlying internal limiting membrane was reapposed to the retinal surface rather than elevated or detached, was of normal caliber and reflectivity, and exerted no discernible tangential traction on the fovea (Figure 2B,D).

With vision improving, no traction and no cystoid change at the edges, the patient was counselled and observed with serial OCT rather than offered vitrectomy.

## 4. Spontaneous Closure

At review on June 8, 2026, the patient reported further improvement. Uncorrected visual acuity in the left eye was 20/40, improving to 20/20 with a manifest refraction of +0.25 − 0.25 × 85 (right eye +0.25 − 0.50 × 80, 20/20; near addition +2.00) (Table 1).

**Table 1:** Clinical timeline.

Date	Interval	VA (OS)	OCT/findings	Management
January 3, 2026	Day 0	CF	Sudden central scotoma; dense premacular haemorrhage involving the fovea (OD 20/25)	Observation
January 14, 2026	2 weeks	CF	Dome‐shaped hyperreflective foveal lesion with dense posterior shadowing; CST 571 *μ*m (OD 223 *μ*m); foveal architecture not assessable	Observation
January 23, 2026	3 weeks	CF	Unchanged; no layering of the haemorrhage	FFA arranged
January 26, 2026	3 weeks	CF	FFA: blockage at the level of the haemorrhage OS; subtle foveal/perifoveal hyperfluorescence; juxtapapillary and ONH staining OU; no macroaneurysm, occlusion or CNV	Observation
March 2, 2026	8 weeks	20/60 (eccentric fixation)	Haemorrhage cleared. Foveal neurosensory dehiscence beneath an intact ILM; ELM and EZ interrupted; CST 272 *μ*m; no traction, no cystoid edges	Observation elected
June 8,2026	22 weeks	20/40 unaided; 20/20 BCVA	Defect closed. Foveal contour restored; CST 257 *μ*m; central hyperreflective bridging column	Observation; no intervention

Abbreviations: BCVA, best‐corrected visual acuity; CF, counting fingers; CST, central subfield thickness; FFA, fluorescein angiography; ONH, optic nerve head.

OCT showed the defect closed. The cavity had resolved and the foveal contour was restored, with central subfield thickness 257 *μ*m. A vertical hyperreflective column ran from the inner retina to the outer retina and retinal pigment epithelium at the site of the former defect, consistent with glial or Müller‐cell repair tissue filling the dehiscence (Figure 2C,E).

The defect had therefore closed within about 3 months of detection and 5 months of onset, with no membranotomy, vitrectomy, gas or drug, and vision recovering from counting fingers to 20/20.

## 5. Discussion

What stands out here is not only an unusual lesion, a dehiscence of the neurosensory retina under an intact ILM, fitting neither the full‐thickness nor the lamellar category, but that it healed on its own to 20/20, where the one published case with the same anatomy only widened and stayed open. Comparing the two is what makes this case useful.

### 5.1. What This Lesion Is, and What to Call It

By the International Vitreomacular Traction Study Group definition, a full‐thickness macular hole runs through every layer from the ILM to the retinal pigment epithelium [11]; ours spared the ILM and so does not qualify, whatever the loss beneath it. Nor does it satisfy the consensus definition of a lamellar macular hole, which requires an irregular foveal contour, a foveal cavity with undermined edges and apparent loss of foveal tissue [12]; our cavity had neither undermined edges nor intraretinal splitting, and the tissue lost was outer rather than inner. Xie et al. met the same difficulty and settled on “outer retinal lamellar macular hole” [10]—a designation Oh et al. later disputed, holding that the loss of photoreceptors at the base made the lesion a full‐thickness hole under International Vitreomacular Traction Study Group criteria [9]. That disagreement is itself telling: neither existing category accommodates this anatomy comfortably. We prefer to describe it plainly, as a foveal neurosensory dehiscence beneath an intact ILM, and think it worth recognising in its own right, since it may behave quite differently from a true hole.

### 5.2. The Diagnosis: Why Valsalva‐Type, and What Else Was Considered

The haemorrhage looked like the premacular bleed a Valsalva manoeuvre produces, abrupt, painless, unilateral, dense and centred on the fovea [1]. However, no manoeuvre could be elicited, and the patient was a middle‐aged hypertensive man, so we do not assert a Valsalva cause.

The main alternative is a ruptured retinal arterial macroaneurysm, which favours hypertensive patients of this age and produces the same multilevel pattern of bleeding, including after a Valsalva manoeuvre [13]. Angiography could not settle this, because the haemorrhage blocked the fovea; what argues against it is the follow‐up. As the blood cleared, no aneurysm, sclerotic arteriole or arterial outpouching appeared at the fovea on fundoscopy or on any of the three OCT scans, and there was no lipid exudation or rebleeding over 5 months. A macroaneurysm also sits on an arcade artery rather than at the foveal centre. Hypertensive retinopathy was absent in the fellow eye, and vein occlusion, choroidal neovascularisation and polypoidal vasculopathy showed none of their angiographic features. A bleeding diathesis was unlikely after a single unilateral episode with no bleeding elsewhere. We cannot claim certainty; a small aneurysm can bleed once and involute before it is ever imaged, so we regard the haemorrhage as spontaneous and of undetermined cause. In any case, how the resulting defect behaved does not depend on why the eye bled.

The faint foveal hyperfluorescence at the edge of the blocked area may have been an early window defect, hinting the dehiscence was already there beneath the blood; with the fovea unreadable we cannot say, and neither could earlier authors [9].

### 5.3. Why Did This Defect Close When the Comparable One Did Not?

This is the substantive question the case poses, and the two cases differ in a way that suggests an answer.

#### 5.3.1. Formation

A sub‐ILM bleed balloons the membrane upward, and as it drains, the stretched ILM pulls back on the fovea, which is thin enough to give way [10]. The ILM is tougher than the tissue beneath it, so that tissue can tear while the membrane holds, just what we and Xie et al. saw. Eight weeks of blood on the fovea may have weakened it further [4, 5, 10]. We also cannot rule out that the fovea gave way at the moment of the bleed and the defect was simply hidden until the blood cleared [10].

#### 5.3.2. Closure, and the Role of the ILM

Idiopathic full‐thickness holes close on their own only rarely, and when they do it is usually the small, early ones without vitreofoveal traction, filled in by glial or Müller‐cell tissue [14]. Our patient fit that profile, and the closure scan shows the mechanism directly: a hyperreflective column runs from inner to outer retina where the defect had been, which is glia filling the gap rather than the edges sliding back together. Vision returned to 20/20, so the photoreceptors must have largely recovered too (Table 2).

**Table 2:** Reported foveal dehiscence and macular hole following premacular haemorrhage of Valsalva or spontaneous type.

Report	Age/sex	Trigger	Intervention	Anatomy of defect	State of the ILM	Outcome	Final VA
Ulbig et al. 1998 [6]	—	Valsalva	Nd:YAG membranotomy	Macular hole identified after laser drainage	Breached by laser	—	—
Kim et al. 2014 [7]	—	Valsalva	Pars plana vitrectomy	Hole developed after vitrectomy	—	Second surgery required	—
Xie et al. 2014 [10]	34/M	Push‐ups	None	Neurosensory discontinuity; outer retinal lamellar hole	Intact, but detached, thickened and tractional	Enlarged 117 → 296 *μ*m; persisted at 17 months	20/25
Suren et al. 2018 [8]	19/M	Push‐ups, sit‐ups	Nd:YAG membranotomy	FTMH 1 week after laser	Breached	Spontaneous closure at 6 months	Improved
Tian et al. 2021 [15]	—/F	Pregnancy	Nd:YAG membranotomy	Macular hole after membranotomy	Breached	Spontaneous closure 12 weeks after laser	—
Gaur et al. 2023 [16]	—	Valsalva	Nd:YAG hyaloidotomy	Parafoveal macular hole after laser	Breached	Spontaneous resolution	—
Oh et al. 2025 [9]	59/M	Straining	None, then PPV + ICG‐assisted ILM peel + gas	Spontaneous FTMH, unmasked at 6 weeks	Reported as still attached over the hole; no PVD or ERM	Failed to close; PPV required; closed 4 weeks post‐op	20/50 post‐op
Present case	51/M	None elicited	None	Neurosensory dehiscence extending to the RPE	Intact, reapposed, normal calibre, no traction	Spontaneous closure by~3 months, with glial bridging	20/20

Abbreviations: FTMH, full‐thickness macular hole; ICG, indocyanine green; ILM, internal limiting membrane; PPV, pars plana vitrectomy.

This is where our case parts company with Xie′s. Their ILM stayed detached, thickened and hyperreflective, and they identified it as still pulling on the fovea, reasoning that a large, long‐standing haemorrhage keeps the membrane too taut to settle back down [10]. Their defect grew from 117 to 296 *μ*m and never closed [10]. Ours was reapposed to the retinal surface, of normal calibre and reflectivity, and exerting no traction, and the defect closed.

The reading we favour is that what happens to a defect like this depends less on its size than on what the ILM does next. Once the membrane settles back onto the retina, the pull that opened the fovea is gone, and the intact ILM gives glia a surface to creep across and close the gap, the same idea behind the inverted‐ILM‐flap repair of large holes [17]. If the ILM stays lifted, thick and taut, it keeps pulling and the defect stays open or widens. Therefore, the preserved ILM cuts both ways: Still detached, it is what held Xie′s defect open; settled back down, it is what let ours heal.

That has a practical corollary, though it must be put carefully. Two earlier holes following Valsalva retinopathy closed without further surgery once the membrane had been opened by Nd:YAG laser [15, 16], so spontaneous repair is not peculiar to our anatomy. What distinguishes the case that failed is more instructive: in the recent spontaneous full‐thickness hole the ILM was described as still attached over the defect, yet the hole neither closed nor improved and vitrectomy with ILM peeling and gas was ultimately required [9]. An adherent membrane is therefore not sufficient in itself. What our case adds is a membrane that was not merely present but reapposed, of normal calibre and exerting no traction. Whether the ILM is intact, and whether it has stopped pulling, may matter more than how large the defect is.

This is a hypothesis from two cases, not an established mechanism, but it is testable: in any future case of this kind, the state of the ILM on the disclosing scan should predict whether the defect closes.

### 5.4. Clinical Implications

A few practical points follow. Improving vision does not rule out a foveal defect; our patient′s acuity had already climbed from counting fingers to 20/60 at the visit where we first saw the hole, simply because the media had cleared and he had taken up eccentric fixation, so a resolving premacular bleed still needs OCT follow‐up. When a defect appears, read the ILM as carefully as the hole: whether the membrane is intact, and whether it has settled back down, tells you what to call the lesion and, we suspect, how it will behave. A defect with the ILM preserved and relaxed, small, recent, without traction or cystoid edges need not go straight to surgery. However, watching cannot be open‐ended; Xie′s defect enlarged, so set criteria in advance for abandoning observation: enlargement, cystoid change, persistent ILM traction, or failure to close within an agreed interval.

### 5.5. Limitations

Several limitations apply. No precipitating manoeuvre could be elicited, so the cause of the haemorrhage rests on morphology and exclusion rather than history, and a macroaneurysm concealed by blood cannot be ruled out with certainty. Calliper measurement was unavailable, so we cannot compare our defect numerically with Xie′s (117 → 296 → 288 *μ*m) or exclude size, rather than the ILM, as the reason for the different outcomes. The presenting OCT was degraded by shadowing and the ILM and posterior hyaloid could not be separately resolved, so we cannot say whether the dehiscence was present at onset or the plane the blood occupied. OCT angiography and microperimetry were unavailable; microperimetry would have documented the eccentric fixation and its resolution objectively. Follow‐up beyond July 2026 is ongoing, so late reopening cannot be excluded. The mechanism we propose rests on a comparison of two cases and needs confirmation in more.

## 6. Conclusion

A foveal neurosensory dehiscence under an intact ILM can appear as a premacular haemorrhage clears and can then heal on its own, restoring vision from counting fingers to 20/20 with no laser or surgery. The one earlier case with this anatomy only enlarged, and there the ILM stayed detached and taut; in ours, it settled back onto the retina and the defect closed. That difference is our proposed explanation, and it carries a simple message: When the ILM is intact and relaxed; this is not automatically a hole for the operating theatre.

**Nomenclature**
BCVA: best‐corrected visual acuity
CF: counting fingers
CST: central subfield thickness
ELM: external limiting membrane
ERM: epiretinal membrane
EZ: ellipsoid zone
FFA: fundus fluorescein angiography
FTMH: full‐thickness macular hole
ILM: internal limiting membrane
IVTS: International Vitreomacular Traction Study Group
OCT: optical coherence tomography
ONH: optic nerve head
PPV: pars plana vitrectomy
PVD: posterior vitreous detachment
RPE: retinal pigment epithelium

## Author Contributions

A.D. and N.J. collected the clinical data, performed the imaging review and drafted the manuscript. Y.T. conceived the report, analysed the serial OCT findings, performed the literature review and revised the manuscript critically for intellectual content. B.B. was responsible for the clinical management of the patient, reviewed, edited and supervised the work.

## Funding

No funding was received for this manuscript.

## Disclosure

All authors have read and approved the final manuscript.

## Ethics Statement

Ethical approval was not required for this single‐case report at the participating institutions. The work was conducted in accordance with the tenets of the Declaration of Helsinki.

## Consent

Written informed consent was obtained from the patient for publication of this case report and all accompanying images. A copy of the signed consent form is available for review by the editor in chief on request.

## Conflicts of Interest

The authors declare no conflicts of interest.

## Supporting information


**Supporting Information** Additional supporting information can be found online in the Supporting Information section.

## Data Availability

The data that support the findings of this study are available from the corresponding author upon reasonable request.
