# Supplementary material for: Spontaneous Closure of a Foveal Neurosensory Dehiscence Beneath an Intact Internal Limiting Membrane Following Spontaneous Premacular Haemorrhage: A Case Report
Source: Case Rep Ophthalmol Med. 2026 Nov 14;2026:4227953. doi: 10.1155/crop/4227953 (PMC13768995; doi:10.1155/crop/4227953)
Supplement: Supplementary file 1 [file CROP-2026-4227953-s001.pdf]

CARE case report guidelines

CARE Checklist of information to include when writing a case report

(cc) BY-NC-ND

Topic	Item	Checklist item description	Reported on Line
Title	1	The diagnosis or intervention of primary focus followed by the words "case report"	1–3
Key Words	2	2 to 5 key words that identify diagnoses or interventions in this case report, including "case report"	34–35
Abstract (no references)	3a	Introduction: What is unique about this case and what does it add to the scientific literature?	11–15
	3b	Main symptoms and/or important clinical findings	16–21
	3c	The main diagnoses, therapeutic interventions, and outcomes	21–27
	3d	Conclusion—What is the main "take-away" lesson(s) from this case?	28–33
Introduction	4	One or two paragraphs summarizing why this case is unique (**may include references**)	36–59
Patient Information	5a	De-identified patient specific information.	61–62
	5b	Primary concerns and symptoms of the patient.	61–62, 67
	5c	Medical, family, and psycho-social history including relevant genetic information	62–66
	5d	Relevant past interventions with outcomes	64–66
Clinical Findings	6	Describe significant physical examination (PE) and important clinical findings.	67–71
Timeline	7	Historical and current information from this episode of care organized as a timeline	137 (Table 1)
Diagnostic Assessment	8a	Diagnostic testing (such as PE, laboratory testing, imaging, surveys).	74–85, 90–104
	8b	Diagnostic challenges (such as access to testing, financial, or cultural)	83–85, 99–100
	8c	Diagnosis (including other diagnoses considered)	95–98, 157–175
	8d	Prognosis (such as staging in oncology) where applicable	115–116, 217–222
Therapeutic Intervention	9a	Types of therapeutic intervention (such as pharmacologic, surgical, preventive, self-care)	105–106
	9b	Administration of therapeutic intervention (such as dosage, strength, duration)	105–106, 115–116
	9c	Changes in therapeutic intervention (with rationale)	105–106, 219–222
Follow-up and Outcomes	10a	Clinician and patient-assessed outcomes (if available)	87–89, 108–110
	10b	Important follow-up diagnostic and other test results	90–104, 111–114
	10c	Intervention adherence and tolerability (How was this assessed?)	105–106, 137
	10d	Adverse and unanticipated events	115–116, 231–232
Discussion	11a	A scientific discussion of the strengths AND limitations associated with this case report	141–144, 223–233
	11b	Discussion of the relevant medical literature **with references**.	146–212
	11c	The scientific rationale for any conclusions (including assessment of possible causes)	157–175, 178–209
	11d	The primary "take-away" lessons of this case report (without references) in a one paragraph conclusion	234–240
Patient Perspective	12	The patient should share their perspective in one to two paragraphs on the treatment(s) they received	Not reported
Informed Consent	13	Did the patient give informed consent? Please provide if requested ... 280–282	Yes ☒ No ☐
